# Hypothiocyanous acid reductase is critical for host colonization and infection by *Streptococcus pneumoniae*

**DOI:** 10.1016/j.jbc.2024.107282

**Published:** 2024-04-09

**Authors:** Heather L. Shearer, Michael J. Currie, Hannah N. Agnew, Claudia Trappetti, Frederick Stull, Paul E. Pace, James C. Paton, Renwick C.J. Dobson, Nina Dickerhof

**Affiliations:** 1 Department of Pathology and Biomedical Science, Mātai Hāora - Centre for Redox Biology and Medicine, University of Otago Christchurch, Christchurch, New Zealand; 2 Biomolecular Interaction Centre, MacDiarmid Institute for Advanced Materials and Nanotechnology and School of Biological Sciences, University of Canterbury, Christchurch, New Zealand; 3 Maurice Wilkins Centre for Molecular Biodiscovery, New Zealand; 4 Department of Molecular and Biomedical Science, Research Centre for Infectious Diseases, University of Adelaide, Adelaide, Australia; 5 Department of Chemistry, Western Michigan University, Kalamazoo, Michigan, USA; 6 Department of Biochemistry and Pharmacology, Bio21 Molecular Science and Biotechnology Institute, University of Melbourne, Parkville, Victoria, Australia

**Keywords:** pneumonia, reductase, *Streptococcu*;, antibiotic resistance, flavoprotein

## Abstract

The major human pathogen *Streptococcus pneumoniae* encounters the immune-derived oxidant hypothiocyanous acid (HOSCN) at sites of colonization and infection. We recently identified the pneumococcal hypothiocyanous acid reductase (Har), a member of the flavoprotein disulfide reductase enzyme family, and showed that it contributes to the HOSCN tolerance of *S. pneumoniae in vitro*. Here, we demonstrate in mouse models of pneumococcal infection that Har is critical for colonization and invasion. In a colonization model, bacterial load was attenuated dramatically in the nasopharynx when *har* was deleted in *S. pneumoniae*. The Δ*har* strain was also less virulent compared to wild type in an invasion model as reflected by a significant reduction in bacteria in the lungs and no dissemination to the blood and brain. Kinetic measurements with recombinant Har demonstrated that this enzyme reduced HOSCN with near diffusion-limited catalytic efficiency, using either NADH (k_cat_/K_M_ = 1.2 × 10^8^ M^−1^s^−1^) or NADPH (k_cat_/K_M_ = 2.5 × 10^7^ M^−1^s^−1^) as electron donors. We determined the X-ray crystal structure of Har in complex with the FAD cofactor to 1.50 Å resolution, highlighting the active site architecture characteristic for this class of enzymes. Collectively, our results demonstrate that pneumococcal Har is a highly efficient HOSCN reductase, enabling survival against oxidative host immune defenses. In addition, we provide structural insights that may aid the design of Har inhibitors.

Antibiotic resistance poses a serious health threat and has been a significant issue worldwide for many years. The Centre for Disease Control and Prevention (CDC) has reported that antimicrobial-resistant infections in the United States increased after the COVID-19 pandemic, with a significant rise in healthcare-associated pathogen infections and deaths, along with an increase in the prescription of antibiotics (1). This highlights the urgent need for new prevention and treatment strategies for priority pathogens.

One strategy for combatting infections is to target bacterial virulence factors, aiding the immune system in eliminating invading pathogens (2, 3, 4). The advantage of this approach is the lower likelihood of driving antibiotic resistance. This is because drugs that target a specific part of the host-pathogen interaction rather than kill the pathogen directly will not exert selection pressure outside the patient. Consequently, the spread of antibiotic-resistant genes through environmental drug exposure is expected to be minimal.

When a pathogen enters a host, multiple defense mechanisms are employed by the immune system. This includes the generation of antimicrobial oxidants by heme peroxidase enzymes lactoperoxidase and myeloperoxidase (5, 6). Key peroxidase-derived oxidants at sites of infection and colonization are hypochlorous acid (HOCl) and hypothiocyanous acid (HOSCN), generated from hydrogen peroxide and chloride or thiocyanate ions, respectively (5, 6, 7). HOSCN is expected to be the main oxidant in epithelial fluids lining the respiratory tract due to high concentrations of thiocyanate at these sites (8, 9, 10, 11, 12). The ability of pathogens to tolerate oxidants produced by the immune system provides a survival advantage. As such, an area of ongoing research is to identify bacterial defense mechanisms against immune-derived oxidative stress in an effort to provide novel antimicrobial targets.

*Streptococcus pneumoniae* is one example of a major human pathogen that is acquiring antibiotic resistance at an alarming rate. Consequently, in 2017 the WHO listed penicillin-non-susceptible *S. pneumoniae* as a priority pathogen, and in 2019 the CDC listed drug-resistant *S. pneumoniae* as a serious threat (13, 14). Furthermore, the Global Burden of Disease study from the Institute for Health Metrics and Evaluation shows that *S. pneumoniae* is causing the majority of deaths resulting from lower bacterial respiratory infections worldwide (15). The impact of *S. pneumoniae* since 2019 cannot yet be determined as updated resistance data has not been reported due to delays in data acquisition as a result of the COVID-19 pandemic (1).

*S. pneumoniae* is a gram-positive bacterium that colonizes the mucosa of the upper respiratory tract, and can cause pneumonia, bacteremia, and meningitis infections, resulting in millions of deaths worldwide. We discovered that *S. pneumoniae* has a high tolerance toward HOSCN (16), which is particularly interesting given that these bacteria produce the precursor for HOSCN, hydrogen peroxide, as part of their normal metabolism (17). This suggests that these bacteria have ways to cope with HOSCN that could be targeted for antimicrobial therapy. We subsequently discovered that *S. pneumoniae* possesses a flavoprotein disulfide reductase, Har, which reduces HOSCN. We showed that Har contributes to pneumococcal HOSCN tolerance *in vitro*, however only when targeted in combination with the glutathione antioxidant system (18). Protection against *in vitro* HOSCN stress was also reported to be conferred by homologous enzymes MerA in *Staphylococcus aureus* and RclA in *Escherichia coli* (19, 20). Whether any of these enzymes enable bacterial survival *in vivo* is unknown.

The reduction of HOSCN by recombinant *E. coli* RclA occurs at near-diffusion controlled rates as demonstrated by kinetic measurements with NADH of k_cat_/K_M_ = 9 × 10^7^ M^−1^s^−1^ (20). Har shares high sequence homology with RclA (47% sequence identity, 64% positives) suggesting that it is likely to have high reactivity towards HOSCN. However, the ability of Har to reduce HOSCN has only been inferred from activity assays using lysates from wild type (WT) and Har deletion strains (Δ*har*) (18). While crystal structures for MerA and RclA exist, confirming their identity as dimeric FAD-containing disulfide reductases (19, 21), Har has not yet been structurally characterized.

The objective of the present study is to demonstrate *in vivo* that Har plays a role in pneumococcal colonization and invasion using mouse models of infection. We also aim to define the kinetic parameters for reduction of HOSCN by Har and report the first Har structure at atomic resolution, data that can be used to design inhibitors of Har function.

## Results

### Har is critical for colonization and invasion in a mouse model of pneumococcal infection

**Figure 1:**
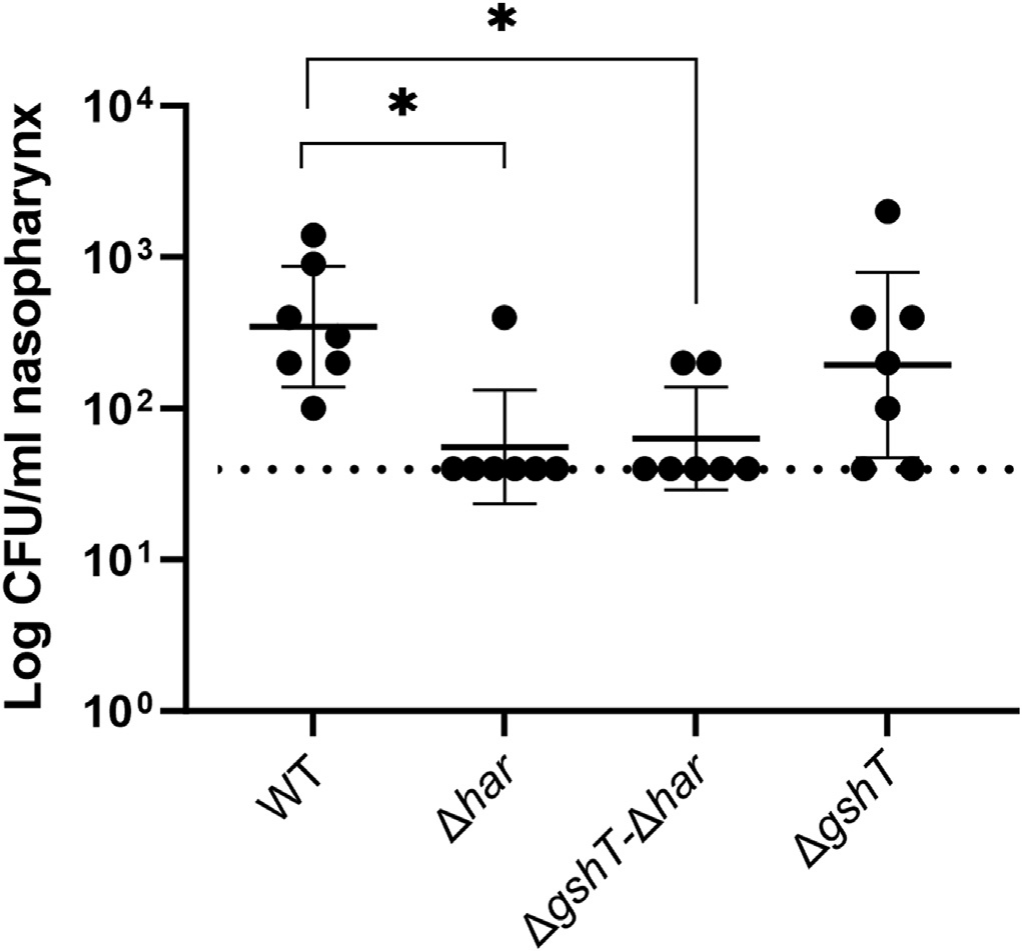
**Nasopharyngeal colonization by *S. pneumoniae* WT, Δ*har*, Δ*gshT*-Δ*har* and Δ*gshT* strains.** Colony-forming units (CFU) recovered in the nasopharynx tissue of mice 24 h following intranasal challenge with 1 × 10^5^ CFU of the respective *S. pneumoniae* strains. The dotted horizontal line denotes the limit of detection. Data are presented as the mean ± SD, with CFU recovered from individual animals shown by symbols. Statistical differences between mutant stains and WT were determined by unpaired two-tailed t-tests and are indicated by ∗ for *p* < 0.05.

A prerequisite for invasive pneumococcal disease is colonization of the upper respiratory tract (22). HOSCN generation is expected to be particularly pronounced in this niche due to the high concentration (up to 800 μM) of the HOSCN precursor thiocyanate (23). To investigate whether Har plays a role in facilitating *S. pneumoniae* nasopharyngeal colonization, we used a colonization murine model in which animals were passively challenged with a small inoculum administered intranasally without anesthesia. In this model, *har* deletion significantly diminished the ability of *S. pneumoniae* to colonize the nasopharynx (Fig. 1), with the number of colony-forming units falling below the limit of quantitation in six out of seven animals (86%). In our previous *in vitro* experiments, sensitization to HOSCN was only observed when the substrate binding protein GshT, which is required for glutathione (GSH) import, was deleted in addition to *har* (18). We therefore included the double-deletion strain Δ*har-*Δ*gshT* in our present *in vivo* investigations. Colonization by the Δ*har-*Δ*gshT* strain was also significantly attenuated compared to the WT strain (Fig. 1). However, this was largely driven by the loss of the reductase, because *gshT* deletion had no impact on its own, and did not curb colonization further when targeted in addition to *har*. These results suggest that the Har activity is important for the ability of *S. pneumoniae* to colonize the nasopharynx, as expected from the levels of HOSCN encountered by the bacteria in this environment.

**Figure 2:**
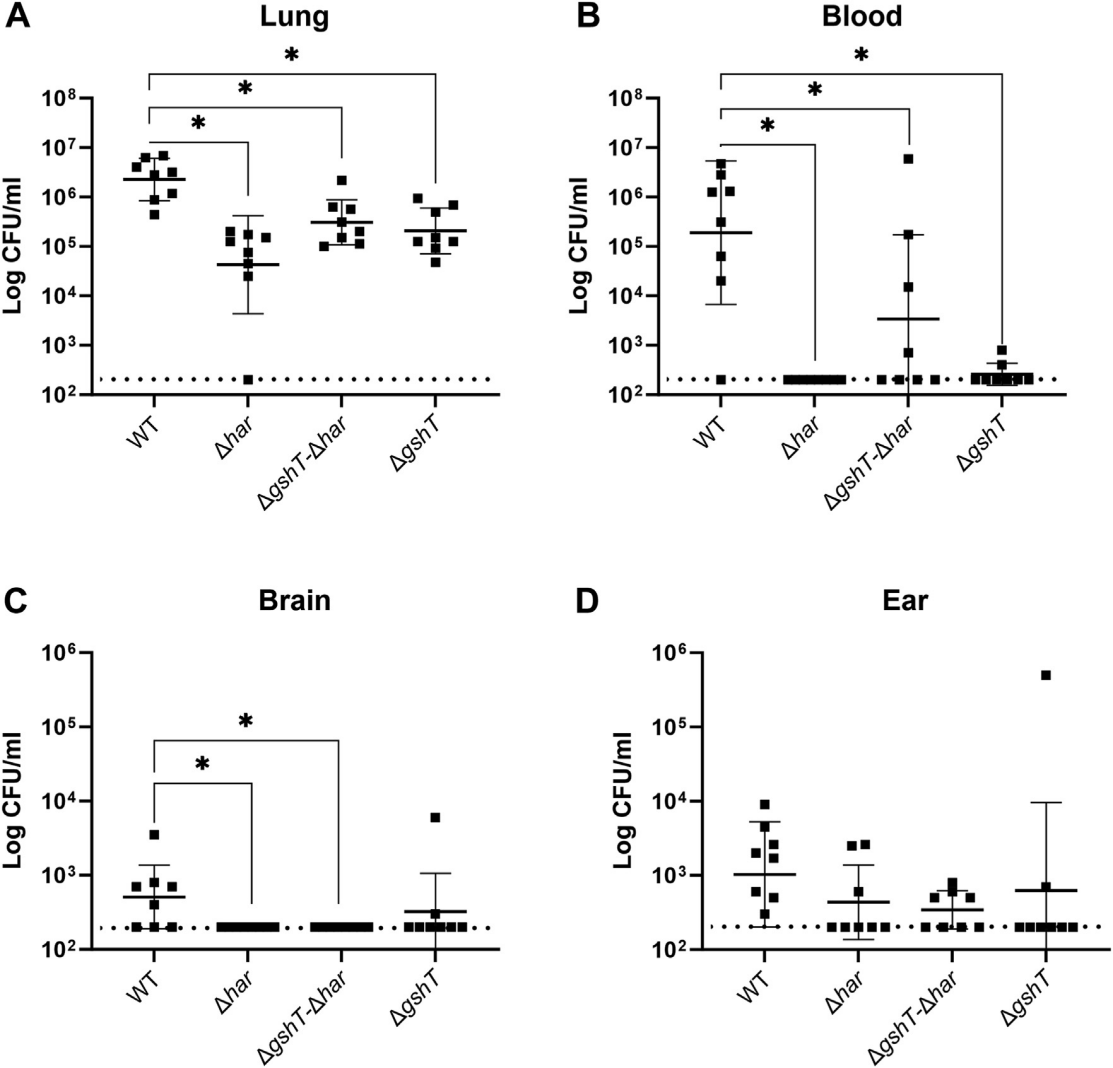
**Invasion by *S. pneumoniae* WT, Δ*har*, Δ*gshT*-Δ*har* and Δ*gshT* strains.** Colony forming units (CFU) recovered from the (*A*) lungs, (*B*) blood, (*C*) brain, and (*D*) ears of mice 24 h following intranasal challenge with 5 × 10^6^ CFU of the respective *S. pneumoniae* strains. The dotted horizontal line denotes the limit of detection. Data is presented as the mean ± SD, with CFU recovered from individual animals shown by symbols. Statistical differences between mutant stains and WT were determined by unpaired two-tailed t-tests and are indicated by ∗ for *p* < 0.05.

Invasive disease occurs when *S. pneumoniae* disseminates to the lung, blood, brain, or inner ears, causing pneumonia, bacteremia, meningitis, or otitis media, respectively (22). We investigated the role of Har in the infection of these sites using a murine invasion model. For this, animals were challenged with a high bacterial load administered intranasally while anesthetized. The number of colony-forming units (CFU) in the lung, blood, brain and ears were determined 24 h post-challenge to assess the extent of pneumococcal invasion. We have previously observed an attenuated phenotype of the Δ*gshT* strain in this model (24). In the present investigation, all three mutant strains Δ*har*, Δ*har*-Δ*gshT* and, in agreement with our previous study, Δ*gshT* showed significantly lower bacterial loads in the lungs compared to the WT strain (Fig. 2*A*). This was mirrored by the diminished ability of these strains to spread to the blood (Fig. 2*B*). Deletion of *har* alone had the greatest effect, resulting in the lowest degree of lung infection and consequently, the blood remained uninfected in all animals challenged with the Δ*har* strain (Fig. 2, *A* and *B*). Only animals that were infected with strains carrying the *har* deletion showed significantly fewer bacteria in the brain when compared to the WT strain (Fig. 2*C*). There were no significant differences between bacterial loads in the ear (Fig. 2*D*). The nasopharynx was not investigated in this model, because it will not accurately reflect colonization due to the high likelihood of re-seeding from the lungs as a result of coughing.

Collectively, our *in vivo* data demonstrates that Har plays an important role in pneumococcal colonization and invasion.

### Har is a highly efficient HOSCN reductase

**Figure 3:**
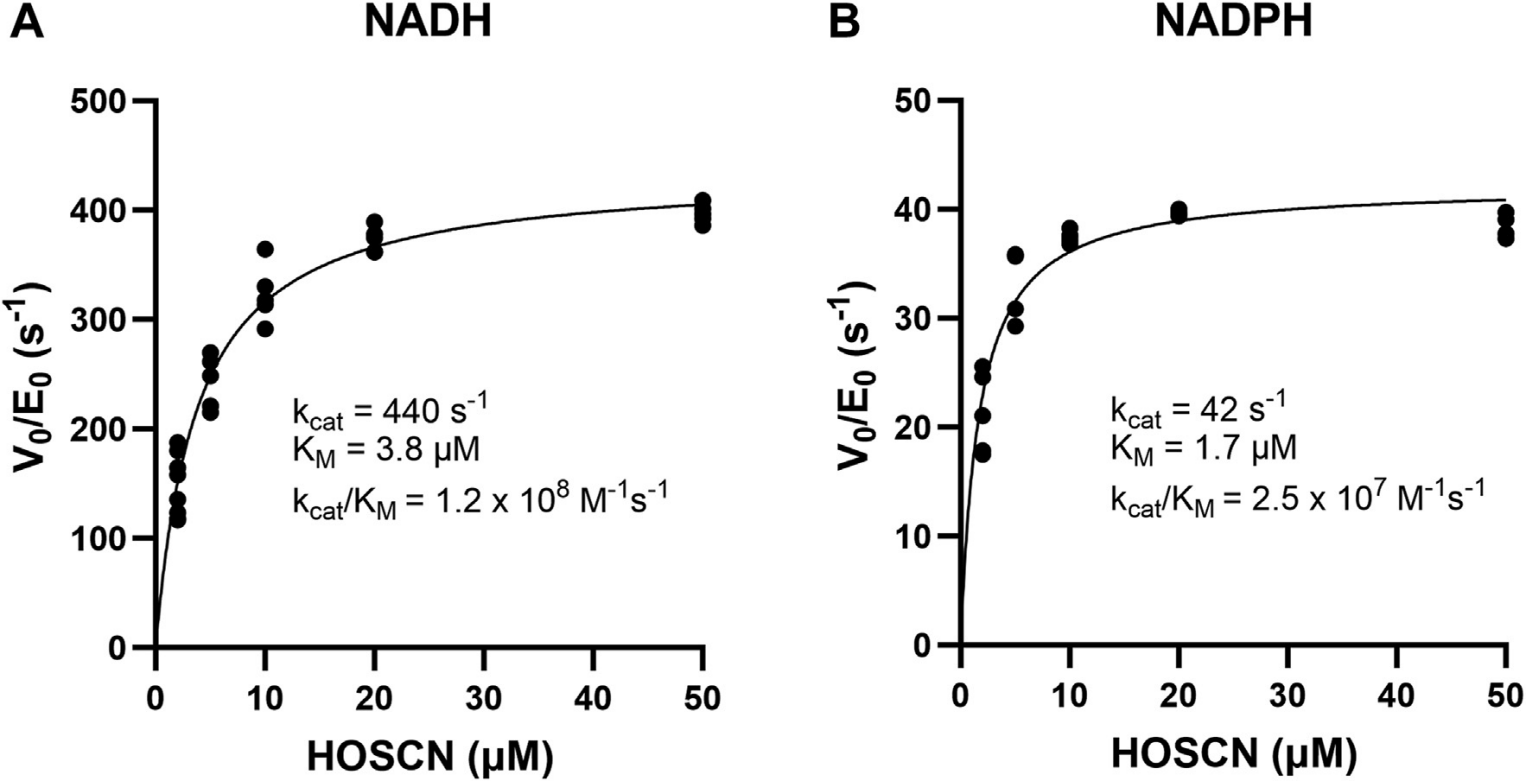
**Michaelis-Menten plots of the rates of NAD(P)H oxidation by Har as a function of HOSCN concentration.** The rates of oxidation of (*A*) NADH (200 μM) and (*B*) NADPH (200 μM) by Har (10 nM) at various concentrations of HOSCN were determined using stopped-flow spectrophotometry.

To determine whether the rate of HOSCN reduction by Har will be relevant *in vivo*, we investigated steady-state kinetic parameters for the enzyme. The rate at which Har consumes NAD(P)H to reduce HOSCN was measured using stopped-flow steady-state kinetics assays as described for the *E. coli* homolog RclA (20). Of the several NAD(P)H concentrations tested, 200 μM was close to the saturation point (data not shown). Like the *E. coli* homolog RclA, Har can utilize both NADH and NADPH as electron donors, but enzyme activity was greater with NADH than with NADPH (Fig. 3). With NADH, Har displayed an apparent second-order rate constant at the diffusion limit of k_cat_/K_M_ of 1.2 × 10^8^ M^−1^s^−1^ resulting from an apparent k_cat_ (turnover number) of 440 ± 10 s^−1^ and an apparent K_M_ (Michaelis constant) of 3.8 ± 0.3 μM (Fig. 3*A*). When utilizing NADPH as the pyridine nucleotide cofactor, the apparent k_cat_ and K_M_ were 42 ± 1 s^−1^ and 1.7 ± 0.2 μM, respectively, resulting in a k_cat_/K_M_ of 2.5 × 10^7^ M^−1^s^−1^ (Fig. 3*B*). Overall, Har reduces HOSCN with kinetic parameters comparable to those reported for the *E. coli* homolog RclA (20), with both enzymes capable of efficiently reducing HOSCN with rate constants near the diffusion-controlled limit.

### Structure of har and comparison to homologous enzymes

**Figure 4:**
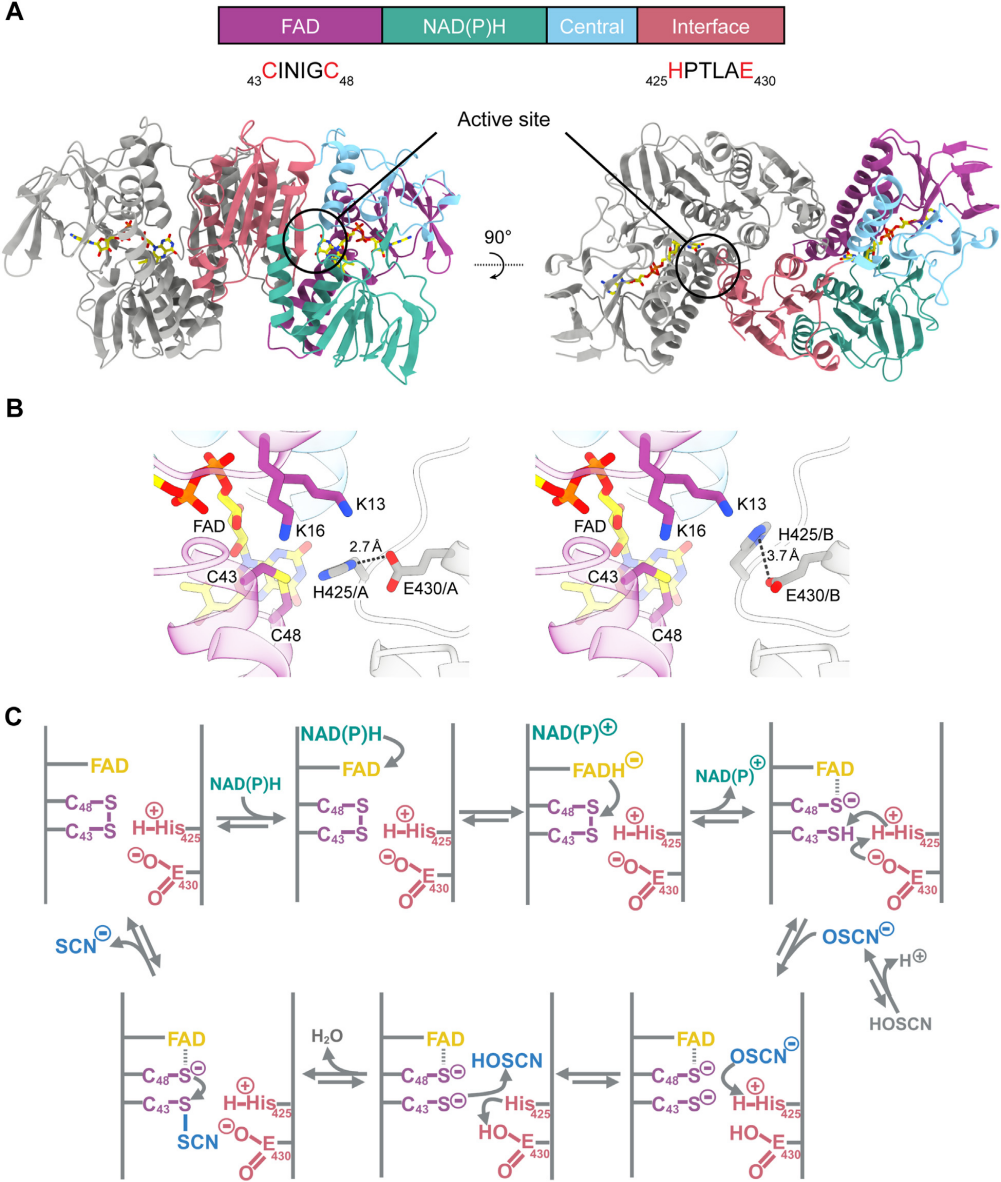
**Crystal structure of Har.***A*, side (*left*) and top (*right*) views of the Har dimer with the four type I flavoprotein disulfide reductase (FDR) domains: FAD, *purple*; NAD(P)H, *green*; central, *blue* and interface, *red*, colored in one monomer. The other monomer is colored *gray*. The bound FAD molecules are shown as *yellow sticks* with standard heteroatom coloring. In the architecture diagram, the two conserved CXXXXC and HXXXXE motifs are shown in the FAD and interface domains. *B*, close-up of the active site residues in Har. The K_13_, K_16_ and catalytic C_43_ and C_48_ residues of the FAD-binding domain (*purple*) are shown as sticks. FAD is shown as *yellow sticks* and heteroatoms are colored as standard (nitrogen, *blue*; oxygen, *red*; sulfur, *yellow*; phosphorous, *orange*). In the structure, H_425_ and E_430_ (*gray sticks*) from the HXXXXE motif were observed in two alternative conformations with equal (50%) occupancy. *Left panel*, one conformation; *right panel*, the alternative conformation. These residues are provided to the active site by the opposing monomer (*gray*). *C*, the proposed mechanism for Har catalysis. Starting from the *top left* and moving clockwise, NAD(P)H donates electrons to the bound FAD cofactor, which reduces the intramolecular disulfide of Har. OSCN^−^ is protonated in the active site before reacting with the catalytic C_43_ residue to form a sulfenylthiocyanate adduct. C_48_ then re-forms the disulfide bond with C_43_ and releases the reduced product thiocyanate SCN^−^. Residues are colored by domain as in *A*. The figure was generated with BioRender.com.

We solved the first crystal structure of full-length Har (438 amino acids + 4 amino acid scar following cleavage of the 6-His tag with HRV-3C protease) with bound FAD (PDB ID: 8UUB, Fig. 4) to a resolution of 1.50 Å, which is the highest resolution obtained for an HOSCN reductase (see Table S1 for data collection and refinement statistics). The model is high quality, with no Ramachandran outliers and good refinement statistics (R_work_ = 15.8%, R_free_ = 18.3%).

Har is a monomer in the asymmetric unit of the crystal but is an expected dimer physiologically as for the homologous enzymes MerA and RclA (19, 21) and other characterized flavoprotein disulfide reductases (25). To verify that Har is a homodimer in solution, sedimentation velocity analytical ultracentrifugation (SV-AUC) was performed on Har at 2 μM and 20 μM (Fig. S1). At both concentrations, a major species (>96% signal) was present at 5.4 S, demonstrating that the sample is monodisperse. The calculated molecular masses of the major species were 99 kDa and 97 kDa for the 2 μM and 20 μM samples, respectively. With a theoretical dimeric mass of 94.9 kDa from the amino acid sequence (Table S3), both samples suggest that Har is present as a homodimer in solution. A minor species (∼2% signal) was present at 4.2 S in the 2 μM sample, which may correspond to a small proportion of monomeric species that exist at lower concentrations. This experiment demonstrates that the single chain in the asymmetric unit of the crystal structure is a result of the crystal symmetry and that Har exists as a homodimer in solution. Furthermore, the frictional ratio (*f/f*_*o*_), a measure of particle asymmetry, was calculated as 1.34 (2 μM) and 1.33 (20 μM) by AUC, identifying a slight asymmetry that is consistent with the *f/f*_*o*_ calculated for the dimeric structure (1.32) by HullRad (26).

The monomer of Har comprises four domains that are characteristic of group I flavoprotein disulfide reductases (FAD, NAD(P)H, central and interface) (Fig. 4*A*) (25). Interestingly, our structure reveals two alternative conformations for the conserved residues H_425_ and E_430_ of the HXXXXE motif in the active site which have not been previously identified. These residues are in the interface domain (Fig. 4*B*) and are known to play an important role in the enzyme’s reaction cycle by acting as an acid/base relay, promoting the formation of a reactive thiolate that subsequently reacts with the substrate (Fig. 4*C*) (25, 27). The C_43_ and C_48_ residues were also solved in two conformations (S-S bound and broken), which could be an artifact whereby exposure to intense X-rays breaks the disulfide bond during data collection. An unexpected area of density was present close to the catalytic C_43_ residue in the active site of the structure, which has been modelled as a malonate dianion (present in the crystallization condition at 1.2 M). The FAD cofactor is bound as in other flavoprotein disulfide reductases, with the isoalloxazine ring positioned adjacent to the catalytic cysteine residues for electron transfer (Fig. 4*B*).

**Figure 5:**
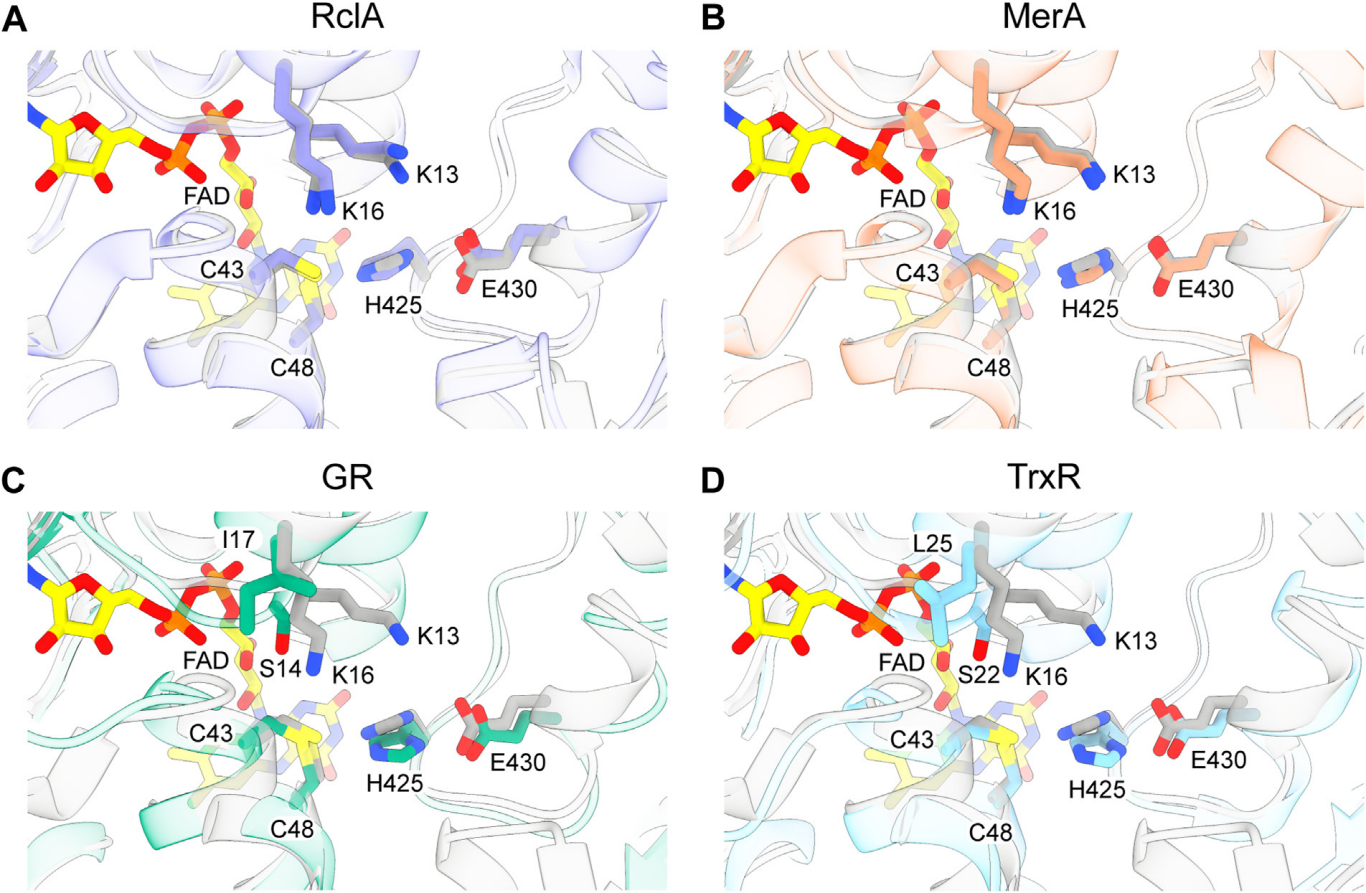
**Overlays of Har with the active sites of similar enzymes showing key residues for substrate binding and catalysis.** Key active site residues C_43_, C_48_, H_425_ and E_430_ (Har numbering) are shown in Har (*gray*) and in (*A*) *E. coli* RclA (6KGY, *purple*) (*B*) *S. aureus* MerA (8AJJ, *orange*) (*C*) *E. coli* glutathione reductase (GR) (1GER, *green*) (*D*) *Homo sapiens* thioredoxin reductase (TrxR) (2ZZC, *blue*). Lysine residues K_13_ and K_16_ are also shown in Har, RclA and MerA, but are absent in *E. coli* GR and *H. sapiens* TrxR, which have S_14_ and I_17_ and S_22_ and L_25_, respectively.

An overlay of homologous HOSCN reductase enzymes *E. coli* RclA, *S. aureus* MerA, and other flavoprotein disulfide reductases, glutathione reductase (GR) and mammalian thioredoxin reductase (TrxR) identified high structural homology among these enzymes (Fig. 5). This is reflected in the root mean square deviation values for these enzymes: RclA (6KGY) = 0.93 Å across 387 C_α_ atoms, MerA (8AJJ) = 0.81 Å across 409 C_α_ atoms, *E. coli* GR (1GER) = 1.06 Å across 319 C_α_ atoms and human TrxR (2ZZC) = 1.14 Å across 282 C_α_ atoms. The key residues for catalysis, *i.e.* the two cysteines, histidine and glutamic acid overlaid across all structures (Fig. 5). However, the active site of Har contains two positively charged lysine residues that are conserved in MerA and RclA, but are absent in GR or mammalian TrxR (Fig. 5).

## Discussion

The present study reveals, for the first time, the crucial role of a member of the recently discovered family of bacterial HOSCN reductase enzymes in causing infection. We show that deletion of this enzyme in the human pathogen *S. pneumoniae* yields an attenuated strain with a greatly diminished capacity for colonization and invasion in mice. The *in vivo* relevance of this class of enzymes is underscored by the exceptionally high catalytic efficiency for the reduction of the immune-derived oxidant HOSCN. Furthermore, our high-resolution crystal structure adds to our understanding of the structural determinants of this activity.

The second-order rate constants measured for Har and elsewhere for RclA for the reaction with HOSCN (20) (1.2 × 10^8^ M^−1^s^−1^ and 9 × 10^7^ M^−1^s^−1^, respectively) suggest that HOSCN is a likely substrate for both enzymes *in vivo*. The only other cellular constituents that react with HOSCN at appreciable rates are other thiol-containing proteins and GSH (8 and 2.5 × 10^4^ M^−1^s^−1^ for cysteine and GSH, respectively) (28, 29). While their reaction with HOSCN is 1000–4000-fold slower compared to Har, their collective concentration is likely going to exceed that of the reductase. GSH alone is present at approximately 4 mM in *S. pneumoniae* (based on the reported 36.4 pmol/10^7^ bacteria (30) and assuming a spherical cell shape with a diameter of 1.25 μM, *i.e*., a volume of 9.6e^−16^ L/cell). Consequently, if cellular Har concentrations are lower than 1 μM, less than 10% of HOSCN will react with Har and the rest will react with GSH. However, this comparison assumes an equal distribution of reactants throughout the cell and Har may in fact be enriched, for example, through biomolecular condensates, near membranes where HOSCN entry into the cell occurs. Also, we have shown that GSH quickly becomes depleted in bacteria exposed to HOSCN (30) and that further protection and recovery mediated by the GSH system will be limited by the rate of reduction of glutathionylated proteins and oxidized glutathione (GSSG) by glutaredoxin and GR, respectively. Therefore, HOSCN degradation by Har will be especially critical during ongoing HOSCN exposure, such as that exerted by the host.

Our observations that Har efficiently breaks down HOSCN and plays a critical role *in vivo* prompts the question of whether *S. pneumoniae* lowers the HOSCN levels at colonization sites. In doing so, *S. pneumoniae* may create favorable conditions for other bacteria susceptible to HOSCN. However, quantifying HOSCN *in vivo* poses significant experimental challenges due to its reactivity towards biomolecules and short half-life, combined with the lack of specific probes or biomarkers for its detection. We have previously measured extracellular HOSCN consumption by *S. pneumoniae* and *S. aureus* expressing the Har homolog MerA *in vitro* (16, 19). HOSCN was readily consumed by media alone with no discernible difference in the oxidant’s half-life in the presence of bacteria. Accelerated loss of HOSCN from the media was observed only when using a MerA-overexpressing *S. aureus* strain, which exhibited a 1000-fold higher level of the reductase enzyme compared to WT (19). At physiological pH, the vast majority of the oxidant exists in its charged, cell-impermeable form hypothiocyanite (OSCN^−^), with less than 1% present as the uncharged HOSCN (pKa = 4.8) (31). Therefore, the observed results were unsurprising since the penetration of cells by HOSCN followed by intracellular consumption would not significantly influence the levels of HOSCN in the extracellular environment. Consequently, we conclude that the protective effect of Har likely stems from its capacity to equip individual cells with the capability to detoxify intracellular HOSCN.

Bacterial HOSCN reductases belong to the family of flavoprotein disulfide reductases that, true to their names, utilize a flavin cofactor to transfer electrons from NAD(P)H to a catalytic disulfide within the active site (25). Upon reduction of the disulfide, one of the resulting active site thiols can interact with the respective substrate, such as glutathione disulfide (GSSG) in the case of GR. The resulting mixed disulfide is then resolved through a nucleophilic attack of the adjacent active site thiol, releasing the reduced product. HOSCN reductases, including Har, MerA, and RclA are expected to form a sulfenylthiocyanate intermediate instead of forming a mixed disulfide (32, 33) (Fig. 4*C*).

Like the bacterial HOSCN reductases, GR and mammalian TrxR can also reduce HOSCN, albeit with at least 1000-fold lower catalytic efficiency (k_cat_/K_M_ of 7.5 × 10^4^ M^−1^s^−1^/5 × 10^3^ M^−1^s^−1^ for *E. coli*/human GR and 2.5 × 10^5^ M^−1^s^−1^ for mammalian TrxR) (20, 34). On the other hand, TrxR from *E. coli* is unable to detoxify HOSCN and is even inhibited by the oxidant (34). Collectively, the kinetic data suggests that among the flavoprotein reductases, only the recently recognized subclass including Har, MerA, and RclA are specialized in reducing HOSCN. This differential capacity to reduce HOSCN can be attributed to structural differences.

**Figure 6:**
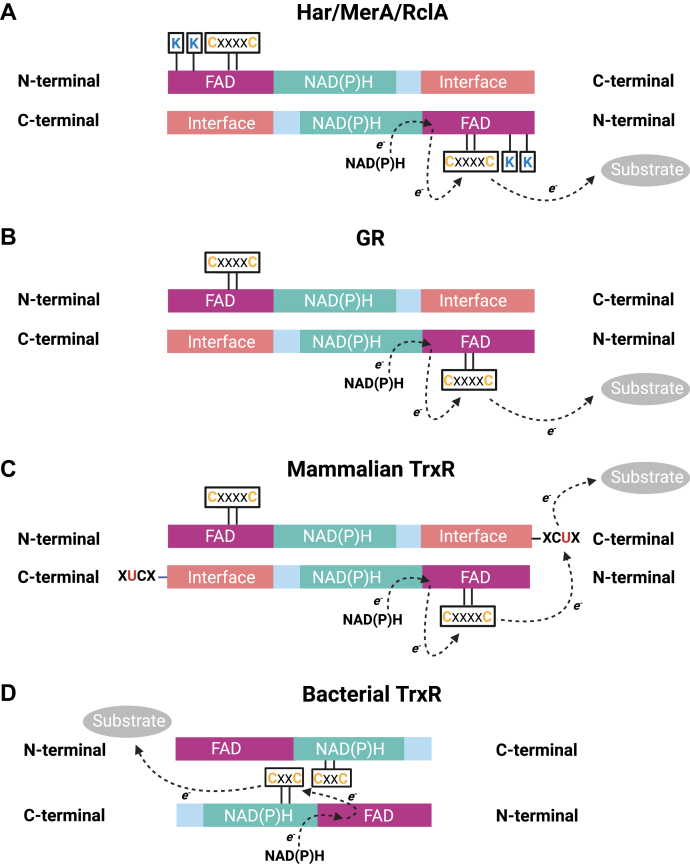
**Domain architecture and catalytic electron transfer of Har in comparison to other flavoprotein disulfide reductases.** To transfer electrons from NAD(P)H to their substrate, (*A*) Har, MerA, RclA, and (*B*) GR use FAD and a catalytic disulfide present in the conserved CXXXXC motif. *C*, Mammalian TrxR contains a C-terminal XCUX motif (U = selenocysteine) as a second non-flavin redox centre, and (*D*) low molecular weight bacterial TrxR (*bottom*) has a CXXC motif that interacts with the substrate following a substantial conformational change. Only the bacterial HOSCN reductases Har, MerA and RclA contain two conserved lysine residues in the active site. The scheme was adapted from (25, 61) and created with BioRender.com.

Bacterial HOSCN reductases have the same domain architecture as GR and mammalian TrxRs (Fig. 6) (19, 21, 25) and here we show that the structures of the active sites surrounding the catalytic CXXXXC motif are highly similar (Fig. 5). Mammalian TrxRs have an additional catalytic selenylsulfide located on a flexible C-terminal to pass electrons to a substrate (Fig. 6) (35, 36). This motif is thought to enable mammalian TrxRs to accept a broader range of substrates. Bacterial TrxRs have a lower molecular weight and a substantially different structural organization (Fig. 6). They lack the interface domain, have a catalytic CXXC motif that is located within the NAD(P)H-binding domain and a conformational shift is required to allow interaction with the thioredoxin substrate (37). A unique feature of the bacterial HOSCN reductases are two positively charged lysine residues in the active site (Fig. 5), which are likely underlying their exceptionally high reactivity towards HOSCN. By providing a positively charged environment, these lysine residues might facilitate interaction with the negatively charged hypothiocyanite anion (OSCN^–^), which is the principal form of the oxidant at physiological pH (31).

While the structural distinctions of these flavoprotein disulfide reductases impact their reactivity and substrate specificity, they offer an opportunity for designing inhibitors. Inhibition of Har could be accomplished by targeting the catalytic cysteine through covalent modification, akin to approaches used for inhibiting mammalian TrxR using selenium compounds, Michael acceptors, and electrophilic nitro aromatic compounds (38). Selectivity over TrxR could be achieved by leveraging electrostatic interactions with the lysine residues in the Har active site. Alternatively, direct targeting of the lysine residues could decrease Har’s reactivity towards its substrate. The crystal structure presented here will serve as a basis for docking-based virtual screening approaches to identify potential lead compounds.

In the present study, we observed that similar to RclA (20), Har prefers NADH over NADPH as the electron donor for the reduction of HOSCN. Reductive processes such as those involving GR and TrxR are generally known to utilize NADPH (39). NAD(H), on the other hand, is recognized as a co-factor in oxidative degradation involving dehydrogenases, such as dihydrolipoamide, alcohol, lactate and glyceraldehyde phosphate dehydrogenases (39). These enzymes reduce NAD^+^ to NADH, which is subsequently used for ATP production.

Certain structural motifs present in the βαβ fold of the nicotinamide nucleotide-binding domain are known to facilitate the molecular distinction between the two co-factors (39). Two arginine residues confer specificity for NADPH by interacting with the negatively charged 2′-phosphate group (39) (Fig. S2). A glycine residue in lieu of alanine in the highly conserved GXGXXG/A region imparts binding to NADH (Table S2). Consistent with a preference for NADH, RclA and Har do not contain the two arginine residues in their nicotinamide nucleotide-binding domain and have the characteristic GXGXXG motif (Table S2, Fig. S2). Other bacterial flavoprotein disulfide reductases with apparent NADH-dependence are also found in other, anaerobic bacteria (40, 41). Lowering the pH can increase NADPH utilization by enzymes with a preference for NADH through protonation of the 2′-phosphate group (39). Whether Har and RclA use NADH or NADPH *in vivo* remains to be determined.

We have previously found that targeting both Har and the GSH antioxidant system in *S. pneumoniae* can completely abolish bacterial growth in the presence of HOSCN generated by lactoperoxidase from pneumococcal-derived H_2_O_2_ (18). In the same model, sensitization to HOSCN was also achieved to some degree by the sole deletion of components of the GSH antioxidant system, while deleting *har* on its own had no effect. Here, we show that not only is deletion of *har* sufficient to decrease bacterial virulence *in vivo*, it had the greatest impact on lowering the bacterial burden in both the colonization and invasion models. The greater importance of Har observed *in vivo* may be due to the bacteria experiencing ongoing HOSCN stress in the nasopharynx and lung, where thiocyanate levels are high, making the capacity for direct oxidant detoxification more advantageous. In contrast, in the *in vitro* system, the HOSCN precursor thiocyanate will eventually be depleted and lactoperoxidase inactivated, allowing for reduction and recovery of cellular components to occur as discussed above. It is also likely that *S. pneumoniae* accumulates less GSH *in vivo* compared to when grown in rich culture. GSH availability in the epithelial lining fluid will be especially compromised during infections when the production of oxidants and hence consumption of the antioxidant will be high (42). A final possibility is that in addition to HOSCN reduction, Har has other as yet undiscovered functions that are important for its interaction with the host.

Unexpectedly, targeting bacterial GSH import along with deleting Har was not associated with a further attenuation in phenotype in the murine infection model. In fact, the double deletion mutant Δ*har*-Δ*gshT* showed a slightly higher bacterial burden in the lungs and blood compared to both of the single mutants. It is conceivable that in response to losing two crucial components of their defense against the immune system, the bacteria upregulated other virulence factors. In support of this proposal are previous observations of compensatory cross-talk between antioxidant systems in *S. pneumoniae* (18, 43).

## Conclusion

The recognition of the bacterial HOSCN reductase as a driver of infection strengthens our understanding of HOSCN as an important component of mammalian immune defense. By demonstrating that Har deletion can lessen pneumococcal colonization and invasive disease, our study endorses Har as a promising target for antimicrobial therapy. Structural characterization of the enzyme is a valuable step for inhibitor design.

## Experimental procedures

### Animal studies

Animal experiments were approved by the University of Adelaide Animal Ethics Committee. For the colonization model, seven female outbred 4- to 6-week-old CD-1 (Swiss) mice per strain were challenged intranasally (without anesthesia) with 10 μl of bacterial suspension containing 1 × 10^5^ CFU in serum broth. The challenge dose was retrospectively confirmed by serial dilution and plating on blood agar. At 24 h post-challenge, animals were euthanized by CO_2_ asphyxiation before harvesting the nasopharynx. Tissues were homogenized and pneumococci enumerated as previously described by serial dilution and plating on blood agar plates containing 5 μg/ml gentamicin (44). Detection limit was 40 CFU.

The murine invasion model was as above, but eight mice per strain were anesthetized by intraperitoneal injection of ketamine (8 mg/ml) and xylazine (0.8 mg/ml), and then challenged intranasally with 50 μl of bacterial suspension containing 5 × 10^6^ CFU in serum broth as previously described (45). At 24 h post-challenge, mice were euthanized by CO_2_ asphyxiation before harvesting the blood, lungs, ears, and brain. Tissues were homogenized and pneumococci were enumerated as above.

### Expression and purification of Har

*E. coli* BL21 (DE3) cells were transformed with the plasmid pET28a-Har (Genscript) containing the Har sequence with an N-terminal 6-His tag and either an HRV-3C tag or Factor Xa cleavage site (Table S3). For all experiments except for stopped-flow kinetics, Har was produced using the HRV-3C construct and the 6-His tag was cleaved off following protein purification, leaving a four amino acid scar at the N-terminal (Table S3).

Cells were cultured in 2 l of LB medium with 50 μg/ml kanamycin at 37 ºC with shaking at 180 rpm until an OD_600_ of 0.6. Protein expression was induced with 1 mM IPTG for 18 h at 25 ºC with shaking at 180 rpm. Cells were harvested by centrifugation at 8000*g* for 6 min, then resuspended in lysis buffer (20 mM Tris-HCl pH 8.0, 500 mM NaCl, and 20 mM imidazole) with 0.5 mg/ml of lysozyme and 500 μM FAD. All purification steps were performed on ice or at 4 °C. Cells were lysed by sonication in 5 min intervals for a total of 15 min then centrifuged at 25,000*g* for 30 min to remove debris. The supernatant was loaded onto a 5 ml HisTrap FF column (Cytiva). After washing with lysis buffer, Har was eluted with elution buffer (20 mM Tris-HCl pH 8.0, 150 mM NaCl, and 250 mM imidazole). For crystallography, Har was diluted until the concentration of imidazole was below 20 mM before 10 mM DTT was added and the purification tag was cleaved by incubation with HRV-3C protease (1:50 w/w protease:Har) overnight at 4 °C. Bio-Rad Nuvia IMAC resin was used to remove the cleaved purification tag, HRV-3C protease, and uncleaved protein from the sample, using a gravity flow column. The cleaved protein was concentrated in 30 kDa molecular weight cutoff spin filters (Amicon, Millipore) and injected onto a HiLoad 16/600 Superdex 200 pg column (Cytiva) equilibrated with 20 mM Tris-HCl pH 8.0, 150 mM NaCl. The purity and molecular weight of the cleaved protein were confirmed using intact protein mass spectrometry (Fig. S3). The protein concentration was determined using the extinction coefficient for FAD (ϵ_450_ = 11,300 M^−1^ cm^−1^).

For stopped-flow kinetic analyses, Har was purified as above with the following modifications: Har containing an N-terminal 6-His tag was purified in one step using a HisTrap column as described above after which it was exchanged into 100 mM sodium phosphate buffer, pH seven using an Econo-Pac 10DG desalting column (Bio-Rad).

### Stopped-flow steady-state enzyme kinetic assays

Kinetic assays were performed using a TgK Scientific SF-61 DX2 KinetAsyst stopped-flow spectrophotometer as described previously (20). The oxidation of 200 μM NADH or NADPH following the addition of 6-His-tagged Har (10 nM) and HOSCN (1–50 μM) in 100 mM sodium phosphate buffer, pH 7 at 25 °C, was monitored by measuring the change of absorbance at 340 nm. The Michaelis-Menten equation was fitted to a plot of initial velocity against the HOSCN concentration using KaleidaGraph and k_cat_ ± S.E. and K_M_ ± S.E. were determined from the fit. Several NAD(P)H concentrations were tested initially and 200 μM was found to be close to the saturation for the reaction velocity. The 6-His tag had no effect on enzyme activity as demonstrated by kinetic measurements using the cleaved enzyme (Fig. S4).

### Har crystallization

Crystal screens (Molecular Dimensions) were set with a Mosquito liquid dispensing robot (SPT Labtech) using the sitting drop vapor diffusion method. Concentrated Har (14.6 mg/ml) was mixed at a 1:1 ratio with reservoir solutions to give 800 nl drop volumes and incubated at 20 °C. Crystals appeared after 3 days in 2.4 M sodium malonate dibasic, pH 7.0 and were frozen in liquid nitrogen without additional cryoprotectant solutions.

### Structure determination

Crystallographic data was collected at the Australian Synchrotron MX2 beamline using an Eiger 16M detector (46). The data were reduced with XDS (47) and scaled with AIMLESS (48, 49). Molecular replacement was performed by MrBUMP (49, 50), which used an AlphaFold (51) structure prediction of UniProt entry Q2G0I4 as the search model. Structure refinement was performed using REFMAC (49, 52) and phenix.refine (53). Iterative improvement of the map and the model was performed using alternate cycles of refinement and residue-by-residue analysis in Coot (49, 54). Data collection and refinement statistics are presented in Table S1. Molecular graphics and analyses were made with UCSF ChimeraX (55).

### Analytical ultracentrifugation

Sedimentation velocity experiments were performed in a Beckman Coulter Optima analytical ultracentrifuge. Har samples at 20 μM (0.95 mg/ml) and 2 μM (0.095 mg/ml) in 20 mM Tris-HCl pH 8.0, 150 mM NaCl were analyzed at 50,000 rpm, 20 °C and 289 nm (20 μM sample) or 231 nm (2 μM sample). Buffer density, buffer viscosity, and protein partial specific volume values were calculated with UltraScan 4.0 (56) (https://github.com/ehb54/ultrascan3). Sedimentation data were analyzed with UltraScan 4.0 (56) (https://github.com/ehb54/ultrascan3). Optimization was performed by two-dimensional spectrum analysis (2DSA) (57, 58) with simultaneous removal of time and radially invariant noise contributions and fitting of boundary conditions. The 2DSA solutions were subjected to parsimonious regularization by genetic algorithm analysis (59).

### Statistical analyses

Statistical significance between log-transformed CFU obtained for mutant strains compared to WT was determined in GraphPad Prism (Version 10.0.2) using unpaired, two-tailed t-tests. *p* values < 0.05 were deemed statistically significant.

## Data availability

Structure coordinates for Har have been deposited in the Protein Data Bank (PDB) under PDB ID 8UUB.

## Supporting information

This article contains supporting information (26, 39, 58, 60).

## Conflict of interest

The authors declare that they have no conflicts of interest with the contents of this article.
