# Supplementary material for: Hypothiocyanous acid reductase is critical for host colonization and infection by Streptococcus pneumoniae
Source: J Biol Chem. 2024 Apr 9;300(5):107282. doi: 10.1016/j.jbc.2024.107282 (PMC11107202; doi:10.1016/j.jbc.2024.107282)
Supplement: Supporting Information [file mmc1.docx]

**Supplementary Material for**

**Hypothiocyanous acid reductase is critical for host colonization and infection by *Streptococcus pneumoniae***

**Heather L Shearer, Michael J Currie, Hannah N Agnew, Claudia Trappetti, Frederick Stull, Paul E Pace, James C Paton, Renwick C J Dobson and Nina Dickerhof**

**This file includes:**

**Table S1.** X-ray crystallography data collection and refinement statistics.

**Table S2.** Amino acid comparison of the βαβ dinucleotide binding fold of dinucleotide-binding enzymes.

**Table S3.** Har protein sequence used in this study.

**Figure S1.** Oligomeric state of Har.

**Figure S2.** Overlay of the dinucleotide binding domains of Har (green) and *H. sapiens* TrxR (2ZZC, blue).

**Figure S3.** Intact mass spectrum of purified Har.

**Figure S4.** The effect of the His purification tag on the HOSCN reductase activity of Har.

**Table S1. X-ray crystallography** **data collection and refinement statistics.** Parentheses indicate statistics for the highest resolution shell.

| Parameters | Har |
| --- | --- |
| Wavelength (Å) | 0.9537 |
| Resolution (Å) | 47.06–1.50 (1.53–1.50) |
| Space group | P4_3_2_1_2 |
| Cell dimensions a, b, c (Å) | 122.26, 122.26, 92.44 |
| Total reflections | 2,951,019 (119,692) |
| Unique reflections | 110,989 (4,979) |
| Multiplicity | 26.6 (24.0) |
| Completeness (%) | 99.6 (91.2) |
| *I*/σ*I* | 15.2 (2.5) |
| Wilson *B*-factor | 18.25 |
| R_merge_ | 0.125 (1.498) |
| R_meas_ | 0.127 (1.530) |
| R_pim_ | 0.025 (0.304) |
| CC_1/2_ | 0.999 (0.754) |
| Reflections in refinement | 110,895 |
| Reflections for R_free_ | 5,590 |
| R_work_ | 0.1579 |
| R_free_ | 0.1830 |
| No. of nonhydrogen atoms | 4,368 |
| Macromolecules | 3,607 |
| Ligand | 60 |
| Solvent | 701 |
| Protein residues | 442 |
| RMS deviations bonds (Å) | 0.014 |
| RMS deviations angles (°) | 2.02 |
| Ramachandran favored (%) | 98.4 |
| Ramachandran allowed (%) | 1.6 |
| Ramachandran outliers (%) | 0 |
| Rotamer outliers (%) | 1.75 |
| Clash score | 6.48 |
| Average B-factor (Å^2^) | 23.04 |
| Macromolecules | 20.69 |
| Ligands | 16.21 |
| Solvent | 35.75 |
| Protein Data Bank entry | 8UUB |

| **Table S2. Amino acid comparison of the βαβ dinucleotide binding fold of dinucleotide-binding enzymes.** Adapted from [39] with the sequences of RclA and Har added. The number denotes the position of the first residue shown for each sequence within each enzyme’s amino acid sequence. Residues highlighted in red and blue are characteristic of NADP(H)- and NAD(H)-utilizing enzymes, respectively. | | | | | | | | | | | | | | | | | | | | | | | | | | | | | | | | | | | | | | | | | | | | | | | | | | | | | | | | | | | | | | | | | | | | | | | | | | | | | | |
| --- | --- | --- | --- | --- | --- | --- | --- | --- | --- | --- | --- | --- | --- | --- | --- | --- | --- | --- | --- | --- | --- | --- | --- | --- | --- | --- | --- | --- | --- | --- | --- | --- | --- | --- | --- | --- | --- | --- | --- | --- | --- | --- | --- | --- | --- | --- | --- | --- | --- | --- | --- | --- | --- | --- | --- | --- | --- | --- | --- | --- | --- | --- | --- | --- | --- | --- | --- | --- | --- | --- | --- | --- | --- | --- | --- | --- | --- | --- |
| **NADP(H) binding** | | | | | | | | | | | | | | | | | | | | | | | | | | | | | | | | | | | | | | | | | | | | | | | | | | | | | | | | | | | | | | | | | | | | | | | | | | | | | |  |
| **Glutathione reductase** | | | | | | | | | | | | | | | | | | | | | | | | | | | | | | | | | | | | | | | | | | | | | | | | | | | | | | | | | | | | | | | | | | | | | | | | | | | | | | |
| *E. coli* | 174 | | **G** | A | | **G** | | | Y | I | | | **A** | V | | | E | | L | | **A** | | G | | V | | I | | N | | G | | L | | **G** | | A | | | K | | | T | | H | | | L | | | F | | | **V** | | **R** | | K | | H | | A | | P | | L | | | **R** | | S | | F | | D | |  | |
| *H. sapiens* | 194 | | **G** | A | | **G** | | | Y | I | | | **A** | V | | | E | | M | | **A** | | G | | I | | L | | S | | A | | L | | **G** | | S | | | K | | | T | | S | | | L | | | M | | | **I** | | **R** | | H | | D | | K | | V | | L | | | **R** | | S | | F | | D | |  | |
| **Thioredoxin reductase** | | |  |  | |  | | |  |  | | |  |  | | |  | |  | |  | |  | |  | |  | |  | |  | |  | |  | |  | | |  | | |  | |  | | |  | | |  | | |  | |  | |  | |  | |  | |  | |  | | |  | |  | |  | |  | |  | |
| *E. coli* | 152 | | **G** | G | | **G** | | | N | T | | | **A** | V | | | E | | E | | **A** | | L | | Y | | L | | S | | N | | I | | A | | S | | | E | | | V | | H | | | L | | | I | | | **H** | | **R** | | R | | - | | D | | G | | F | | | **R** | | A | | E | | K | |  | |
| *H. sapiens* | 197 | | **G** | A | | **S** | | | Y | V | | | **A** | L | | | E | | C | | **A** | | G | | F | | L | | A | | G | | I | | **G** | | L | | | D | | | V | | T | | | V | | | M | | | **V** | | **R** | | S | | - | | I | | L | | L | | | **R** | | G | | F | | D | |  | |
| **Trypanothione reductase** | | |  |  | |  | | |  |  | | |  |  | | |  | |  | |  | |  | |  | |  | |  | |  | |  | |  | |  | | |  | | |  | |  | | |  | | |  | | |  | |  | |  | |  | |  | |  | |  | | |  | |  | |  | |  | |  | |
| *T. congolese* | 195 | | **G** | G | | **G** | | | F | I | | | S | V | | | E | | F | | **A** | | G | | I | | F | | N | | A | | Y | | **G** | | G | | | K | | | V | | T | | | L | | | C | | | **Y** | | **R** | | N | | N | | P | | I | | L | | | **R** | | G | | F | | D | |  | |
| **Mercuric reductase** |  | |  |  | |  | | |  |  | | |  |  | | |  | |  | |  | |  | |  | |  | |  | |  | |  | |  | |  | | |  | | |  | |  | | |  | | |  | | |  | |  | |  | |  | |  | |  | |  | | |  | |  | |  | |  | |  | |
| *P. aeruginosa* | 277 | | **G** | S | | **S** | | | V | V | | | **A** | L | | | E | | L | | **A** | | Q | | A | | F | | A | | R | | L | | **G** | | S | | | K | | | V | | T | | | V | | | L | | | **A** | | **R** | | N | | T | | L | | F | | F | | | **R** | | - | | E | | D | |  | |
|  | | | | | | | | | | | | | | | | | | | | | | | | | | | | | | | | | | | | | | | | | | | | | | | | | | | | | | | | | | | | | | | | | | | | | | | | | | | | | | |
| **NAD(H) binding** | | | | | | | | | | | | | | | | | | | | | | | | | | | | | | | | | | | | | | | | | | | | | | | | | | | | | | | | | | | | | | | | | | | | | | | | | | | | | | |
| **Dihydrolipoamide dehydrogenase** | | | | | | | | | | | | | | | | | | | | | | | | | | | | | | | | | | | | | | | | | | | | | | | | | | | | | | | | | | | | | | | | | | | | | | | | | | | | | | |
| *E. coli* | 180 | **G** | | | G | | **G** | I | | | L | **G** | | | L | E | | M | | **G** | | T | | V | | Y | | H | | A | | L | | **G** | | S | | | Q | | | I | | D | | | V | | V | | | | **E** | | **M** | | F | | D | | Q | | V | | I | | **P** | | | A | | A | | D | |  | | |
| *H. sapiens* | 220 | **G** | | | A | | **G** | V | | | I | **G** | | | V | E | | L | | **G** | | S | | V | | W | | Q | | R | | L | | **G** | | A | | | D | | | V | | T | | | A | | V | | | | **E** | | **F** | | L | | G | | H | | V | | G | | **G** | | | V | | G | | I | | D | | |
| *S. cerevisiae* | 209 | **G** | | | G | | **G** | I | | | I | **G** | | | L | E | | M | | **G** | | S | | V | | Y | | S | | R | | L | | **G** | | S | | | K | | | V | | T | | | V | | V | | | | **E** | | **F** | | Q | | P | | Q | | I | | G | | **A** | | | S | | M | | D | |  | | |
| **HOSCN reductase** |  |  | | |  | |  |  | | |  |  | | |  |  | |  | |  | |  | |  | |  | |  | |  | |  | |  | |  | | |  | | |  | |  | | |  | |  | | | |  | |  | |  | |  | |  | |  | |  | |  | | |  | |  | |  | |  | | |
| *E. coli* (RclA) | 165 | **G** | | | G | | **G** | Y | | | I | **G** | | | V | E | | F | | **A** | | S | | M | | F | | A | | N | | F | | **G** | | S | | K | | | V | | | T | | I | | | | L | | **E** | | | **A** | | A | | S | | L | | F | | L | | | **P** | | R | | E | | D | |  |  |  |
| *S. pneumoniae* (Har) | 164 | **G** | | | G | | **G** | N | | | I | **G** | | | L | E | | F | | **A** | | G | | L | | Y | | N | | K | | L | | **G** | | S | | K | | | V | | | T | | V | | | | L | | **D** | | | **T** | | L | | D | | T | | F | | L | | | **P** | | R | | A | | E | |  |  |  |

**Table S3. Har protein sequence used in this study.**

| **Protein sequence used for crystallography and AUC** (uncleaved mass 52023 Da, cleaved mass 47473 Da) |
| --- |
| MGSSHHHHHHSSGLVPRGSHMASMTGGQQMGRGSEFLEVLFQGPGSMLTYDLIVIGFGKAGKTLAGKLASAGKKVALVERSKAMYGGTCINIGCIPTKTLLVAAEKDLSFEEVIATKNTITGRLNGKNYTTVAGTGVDIFDAEAHFLSNKVIEIQAGDEKQELTAETIVINTGAVSNVLPIPGLATSKNVFDSTGIQSLDKLPEKLGVLGGGNIGLEFAGLYNKLGSKVTVLDTLDTFLPRAEPSIAALAKQYLEEDGIELLQNIHTTEIKNDGDQVLVVTEDETYRFDALLYATGRKPNVEPLQLENTDIELTERGAIKVDKHCQTNVPGVFAVGDVNGGLQFTYISLDDFRVVYSYLAGDGSYTLEDRLNVPNTMFITPALSQVGLTESQAADLKLPYAVKEIPVAAMPRGHVNGDLRGAFKAVVNTETKEILGASIFSEGSQEIINIITVAMDNKIPYTYFTKQIFTHPTLAENLNDLFAI |
| **Protein sequence used for stopped flow kinetics** (uncleaved mass 51451 Da) |
| MGSSHHHHHHSSGLVPRGSHMASMTGGQQMGRGSEFIEGRMLTYDLIVIGFGKAGKTLAGKLASAGKKVALVERSKAMYGGTCINIGCIPTKTLLVAAEKDLSFEEVIATKNTITGRLNGKNYTTVAGTGVDIFDAEAHFLSNKVIEIQAGDEKQELTAETIVINTGAVSNVLPIPGLATSKNVFDSTGIQSLDKLPEKLGVLGGGNIGLEFAGLYNKLGSKVTVLDTLDTFLPRAEPSIAALAKQYLEEDGIELLQNIHTTEIKNDGDQVLVVTEDETYRFDALLYATGRKPNVEPLQLENTDIELTERGAIKVDKHCQTNVPGVFAVGDVNGGLQFTYISLDDFRVVYSYLAGDGSYTLEDRLNVPNTMFITPALSQVGLTESQAADLKLPYAVKEIPVAAMPRGHVNGDLRGAFKAVVNTETKEILGASIFSEGSQEIINIITVAMDNKIPYTYFTKQIFTHPTLAENLNDLFAI |

N-terminal purification tag (underlined), residues in red were cleaved for crystallography and AUC. Har protein sequence from *S. pneumoniae* strain D39 (Genbank: CP000410.2).

**Figure S1. Oligomeric state of Har.** Following purification and cleavage of the purification tag with HRV-3C protease, Har was subjected to sedimentation velocity analytical ultracentrifugation demonstrating that the enzyme is a homodimer in solution. A major species is present at 5.4 S with concentrations of 2 µM (pink) and 20 µM (black). The corresponding molecular weights of these species are consistent with a homodimer. The frictional ratio (*f/f_o_*), which is a measure of particle asymmetry, was also fitted to the data using UltraScan 4.0 (58). The fitted values were 1.34 (2 µM) and 1.33 (20 µM), consistent with an asymmetrical globular protein and led to estimated masses of 99 kDa (2 µM) and 97 kDa (20 µM). These masses are consistent with the dimer mass of 94.9 kDa based on the amino acid sequence (**Table S3**). The fitted *f/f_o_* values from UltraScan 4.0 (1.34 and 1.33) are also consistent with that calculated by HullRad (26) for the dimeric crystal structure presented in this work (1.32).

**
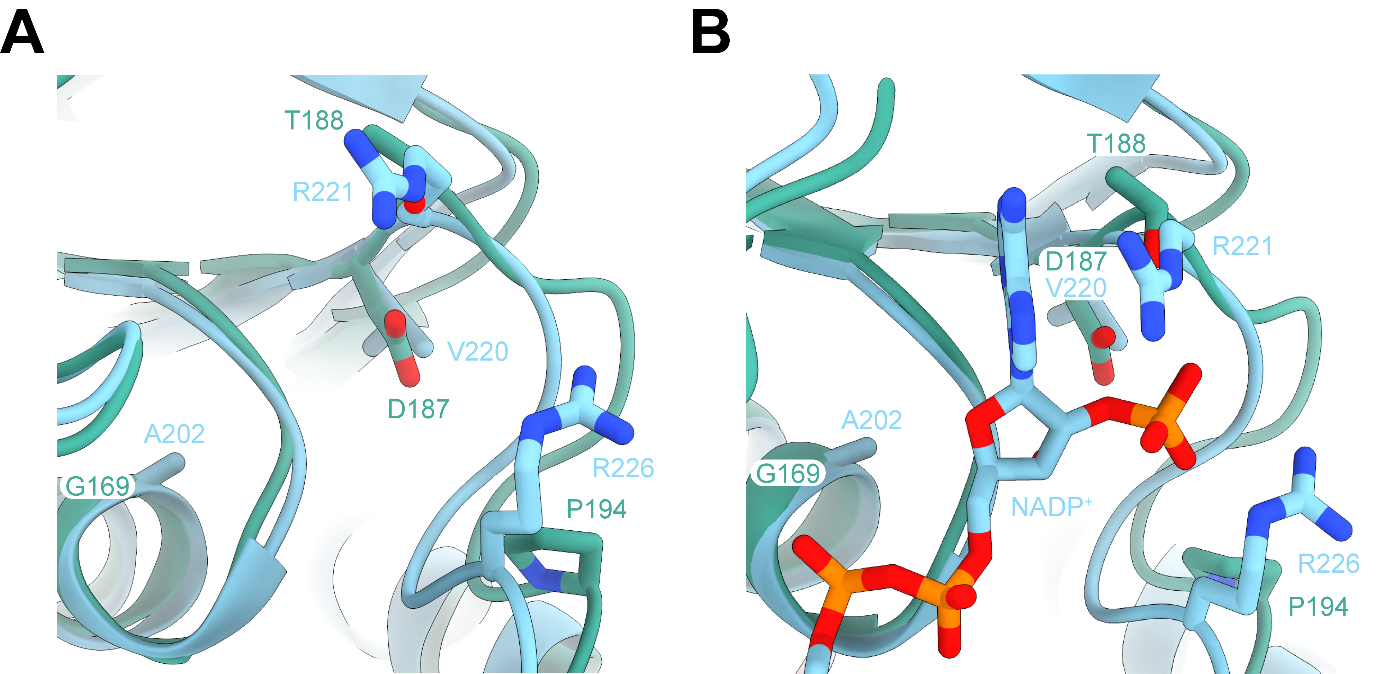
**

**Figure S2. Overlay of dinucleotide binding domains of Har (green) and *H. sapiens* TrxR (2ZZC, blue)**. **A)** The 2’-phosphate group-coordinating arginine residues R_221_ and R_226_ of TrxR are not present in Har, instead T_188_ and P_194_ are present at these positions. **B)** As in **A**, with bound NADP^+^ from the TrxR structure shown, highlighting the position of the 2’-phosphate group between the two arginine residues. V_220_ of TrxR represents a hydrophobic residue conserved at this position in NADP(H) binding sites (**Table S2**), while a negatively charged residue such as D_187_ occupies this site in NAD(H) binding sites. A glycine residue (Gly_169_ of Har) in lieu of alanine (A_202_ of TrxR) in the highly conserved GXGXXG/A region is characteristic of NADH binding (**Table S2**).

**Figure S3. Intact mass spectrum of purified Har.** Following purification and cleavage of the purification tag with HRV-3C protease, Har (0.7 µg) was analyzed by intact LC-MS using a Velos Pro ion trap (Thermo Fisher Scientific) as described before (60). The deconvoluted mass spectrum shows a major peak at 47477 Da, consistent with the theoretical mass of 47473 Da (+/- 5 Da mass accuracy) calculated from the protein sequence (**Table S3**).


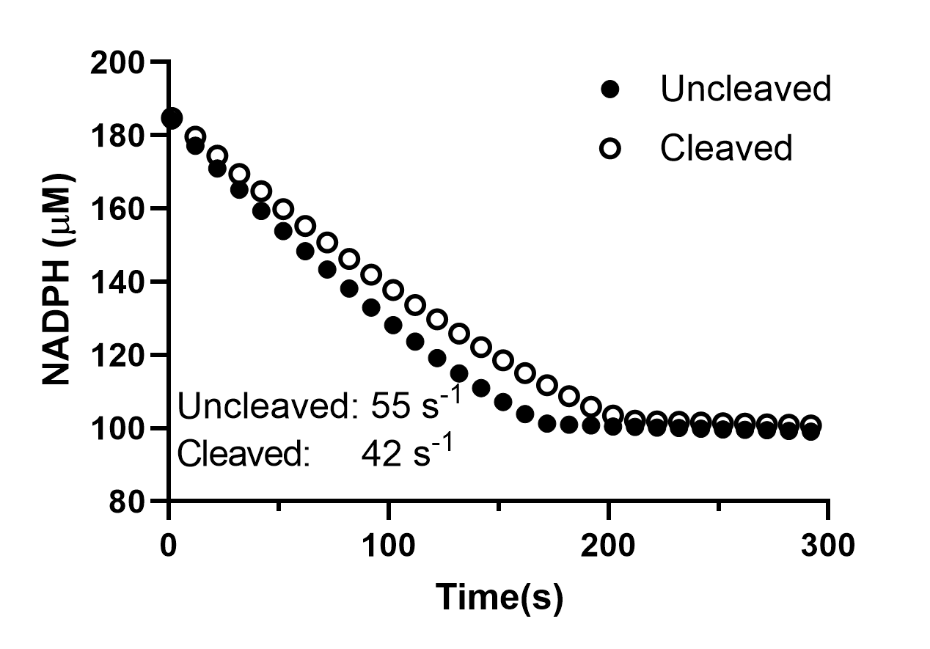


**Figure S4. The effect of the His purification tag on the HOSCN reductase activity of Har.** Recombinant Har containing an N-terminal 6-His tag with an HRV-3C cleavage site was purified as described in Experimental Procedures and the 6-His tag was cleaved with HRV-3C protease. Consumption of NADPH (200 µM) was measured following the addition of either cleaved or His-tagged Har (10 nM) and HOSCN (100 µM) in 100 mM sodium phosphate buffer, pH 7 at 25 °C, by monitoring the loss of absorbance at 340 nm over 5 min in a 1 ml cuvette using an UV-visible spectrophotometer (Agilent 8453, Santa Clara, CA, USA).
